# Functional study and pathogenicity classification of *PRRT2* missense variants in *PRRT2*‐related disorders

**DOI:** 10.1111/cns.13147

**Published:** 2019-05-23

**Authors:** Shao‐Yun Zhao, Li‐Xi Li, Yu‐Lan Chen, Yi‐Jun Chen, Gong‐Lu Liu, Hai‐Lin Dong, Dian‐Fu Chen, Hong‐Fu Li, Zhi‐Ying Wu

**Affiliations:** ^1^ Department of Neurology and Research Center of Neurology in Second Affiliated Hospital, and Key Laboratory of Medical Neurobiology of Zhejiang Province Zhejiang University School of Medicine Hangzhou China

**Keywords:** missense variants, pathogenicity classification, protein level, *PRRT2*, subcellular localization

## Abstract

**Aims:**

*PRRT2* variants are associated with various paroxysmal disorders. To date, more than 90 *PRRT2* variants have been reported in *PRRT2*‐related disorders. Lack of functional study in majority of missense variants makes their pathogenicity uncertain. We aim to evaluate the clinical significance of *PRRT2* missense variants by performing *in vitro* experiments.

**Methods:**

We systematically reviewed *PRRT2*‐related disorders and summarized reported *PRRT2* missense variants. Protein expression and subcellular localization of mutant PRRT2 were investigated in mammal cells. American College of Medical Genetics and Genomics (ACMG) guidelines were used to analyze the pathogenicity of *PRRT2* missense variants.

**Results:**

A total of 29 *PRRT2* missense variants were identified in *PRRT2*‐related disorders. Ten variants were observed to affect both subcellular localization and protein level, three variants only affect membrane localization, and two variants only affect protein level. According to ACMG guidelines, 15 variants were finally classified as “likely pathogenic”, three as “benign”, three as “likely benign”, and eight as “uncertain significance” variants. The likely pathogenic variants were concentrated in the C‐terminal of PRRT2.

**Conclusions:**

The pathogenicity of eight uncertain significance variants needs further investigation. C‐terminal of PRRT2 is crucial for its physiological function.

## 1 INTRODUCTION

*PRRT2* was recently identified as a causative gene of paroxysmal kinesigenic dyskinesia (PKD) and other paroxysmal disorders, including benign familial infantile seizures (BFIS), infantile convulsions with choreoathetosis (ICCA), and paroxysmal hypnogenic dyskinesia (PHD).^1^, ^2^, ^3^, ^4^ Among these *PRRT2*‐related disorders, PKD is the most common phenotype, which is a dominantly hereditary disorder characterized by short and recurrent attacks triggered by a sudden initiation or alteration of voluntary movement.^5^ Age at onset (AAO) is usually during childhood or early adulthood. The primary manifestations are chorea or dystonia with duration <1 minute and a variable frequency ranging from one per month to hundreds per day. Incomplete penetrance is usually observed in *PRRT2* variant carriers.^6^, ^7^

The potential mechanisms of *PRRT2* variants in *PRRT2*‐related disorders remain largely unclear. *PRRT2* consists of four exons and encodes a 340‐amino‐acid protein with two predicted transmembrane (TM) domains in the C‐terminal and one proline‐rich domain in the N‐terminal. Enriched in cerebral cortex, cerebellum, substantia nigra, and hippocampus, PRRT2 protein was found to involve in synaptic transmission by modulating soluble N‐ethylmaleimide sensitive factor attachment protein receptor (SNARE) complex.^8^ To date, more than 90 variants in *PRRT2* have been included in human gene mutation database (HGMD) and most of them are nonsense or frameshift variants, causing the truncation of the protein. Among them, c.649dupC (p.R217Pfs*8) is the most frequent variant, accounting for 78.5% of mutation carriers.^9^ Truncated variants leading to conspicuous reduced protein level have been reported in various *in vitro* studies.^1^, ^3^, ^8^, ^10^ Besides the truncated variants, about 29 missense variants of *PRRT2* are documented in *PRRT2*‐related disorders. However, only three of them has been functionally studied.^11^, ^12^, ^13^ Therefore, the clinical significance of these alleles of *PRRT2* in paroxysmal disorders is difficult to evaluate. It is of great value to perform the functional studies to address the pathogenicity of *PRRT2* missense variants.

In this study, we summarized the reported missense variants in *PRRT2* and performed functional experiments to investigate the alternation of subcellular location and protein expression of *PRRT2* missense variants. We further assigned the pathogenicity of the missense variants according to the guidelines of American College of Medical Genetics and Genomics (ACMG).^14^

## 2 MATERIALS AND METHODS

### 2.1 Missense variants in *PRRT2*

To identify all the reported missense variants within *PRRT2*, we searched the HGMD (http://www.hgmd.org) that provided the most up‐to‐date version of *PRRT2* mutation and the PubMed (https://www.ncbi.nlm.nih.gov/pubmed) from November 2011 when *PRRT2* was first reported as a disease‐causing gene and up to December 2017. The genotype‐phenotype correlation analysis was carried out after a comprehensive literature review. Three in silico tools, SIFT (http://sift.jcvi.org/), PolyPhen‐2 (http://genetics.bwh.harvard.edu/pph2/) and MutationTaster (http://www.mutationtaster.org/), were applied to predict the functional impact of the missense variants. Population databases including 1000 Genomes Project (1000G) (http://browser.1000genomes.org) and Exome Aggregation Consortium (http://exac.broadinstitute.org/) were used to obtain the frequency of variants in populations. The frequency was also evaluated in 2800 healthy individuals of Chinese ancestry (Data from Novogene Company). After that, the pathogenicity of *PRRT2* missense variants was preliminarily evaluated according to the ACMG guidelines.

### 2.2 Plasmid constructs

GenBank sequence NG_032039.1 was used as reference for the *PRRT2* gene, while reference sequences NM_145239.2 and NP_660282.2 were used for *PRRT2* cDNA and protein, respectively. Based on homologous recombination technology, the 1023‐bp *PRRT2* open reading frame was cloned into HindIII/KpnI site of pEGFP‐C2 vector using ClonExpress II One Step Cloning Kit (Vazyme). After that, the variants were introduced by PCR‐mediated mutagenesis with KOD DNA polymerase (Toyobo), followed by confirmation using Sanger sequencing. The primers for mutagenesis plasmids were provided in Table S1.

### 2.3 Cell culture and transient transfection

HEK293T cells and HeLa cells were cultured in DMEM (HyClone) supplemented with 10% fetal bovine serum (Gibco) in a 5% CO_2_ incubator at 37°C. Cells were seeded in suitable wells the day before transfection. Transient transfection was performed using the Lipofectamine 3000 according to the manufacture's protocol (Invitrogen). Forty‐eight hours of cultivation was needed for the protein expression after transfection.

### 2.4 Western blot analysis

To get the protein lysate, cells were rinsed with phosphate buffer saline (PBS) and harvested in lysis buffer. After centrifuging, the supernatants were collected. Western blot was performed as previously described.^15^ The GFP (1:5000) and β‐actin (1:5000; Sigma‐Aldrich) antibodies were used. The blots were semiquantified by gel densitometry using the Photoshop.

### 2.5 Live cell imaging

Growing in 35 mm glass‐bottomed dishes (Shengyou Biotechnology), HeLa cells were transfected with wild‐type or mutant PRRT2 expression plasmids. Forty‐eight hours after transfection, NucBlue Live Reagent (Thermo Fisher Scientific) and Alexa Fluor 594 wheat germ agglutinin (WGA; Invitrogene) were added and incubated for 20 and 10 minutes, respectively. After washing, cells were directly visualized under a confocal microscope (Leica TCS SP8; Leica Microsystems). The green‐fluorescent IOD (integrated optic density) of the whole cell and the cytoplasm was measured by ImageJ.

### 2.6 Statistical analyses

All the experiments were repeated at least three times independently. For the Western blot, proteins were normalized to β‐actin. Differences in the mean values between wild‐type or mutant PRRT2 were analyzed by one‐way ANOVA using GraphPad Prism software. *P* value <0.05 was considered statistically significant.

## 3 RESULTS

### 3.1 Overview of the missense variants in *PRRT2*

After a systematic review of *PRRT2*‐related disorders, we found a total of 29 missense variants in the literature (Table 1). Of which, 26 were heterozygous, two (p.P279L and p.R311W) were homozygous, and one (p.G305R) was described in both homozygous and heterozygous condition.^16^ The predominant phenotype of these missense variants was PKD, while BFIS and ICCA were also documented.

**Table 1:** The profile and classification of the missense variants in *PRRT2*

Variant	Phenotype	1000G	ExAC	Control	SIFT	Polyphen‐2	MutationTaster	Classification^a^	Function^b^	Final Classification
c.412C>G (p.P138A)	PKD, FS+, DS, GEFS+, BECTS	0.03	0.01742	0.071277	Tolerated	Benign	Polymorphism	Benign	Normal	Benign
c.439G>C (p.D147H)	PKD, GEFS+, BECTS	0.003	0.0009088	0.009023	Tolerated	Benign	Polymorphism	Benign	Normal	Benign
c.529G>A (p.E177K)	ID	0	0	0	Deleterious	Benign	Polymorphism	US	Normal	US
c.623C>A (p.S208Y)	DS	0.0002	9.61E‐05	0.000354	Tolerated	Benign	Polymorphism	US	Normal	Likely benign
c.640G>C (p.A214P)	PKD, PHD	0	0.001515	0.003715	Tolerated	Probably damaging	Polymorphism	US	Normal	Likely benign
c.644C>G (p.P215R)	PKD	0.001	0.0007764	0	Tolerated	Probably damaging	Disease causing	US	Normal	Likely benign
c.647C>T (p.P216L)	PKD	0.002	0.007705	0.000534	Deleterious	Possibly damaging	Disease causing	Benign	Normal	Benign
c.647C>G (p.P216R)^c^	PKD	0	0.0001109	0	Deleterious	Possibly damaging	Disease causing	US	Normal	US
c.647C>A (p.P216H)	PKD	0.001	0.0003832	0.000356	Deleterious	Probably damaging	Disease causing	US	Normal	US
c.796C>T (p.R266W)	PKD	0	8.28E‐06	0	Deleterious	Probably damaging	Disease causing	US	Normal	US
c.797G>A (p.R266Q)	PKD	0	1.66E‐05	0	Deleterious	Probably damaging	Polymorphism	US	Normal	US
c.824C>T (p.S275F)	PKD, BFIS	0	0	0	Deleterious	Probably damaging	Disease causing	US	Abnormal	Likely pathogenic
c.836C>T (p.P279L)^d^	BFIS, movement disorder	0	0	0	Deleterious	Probably damaging	Disease causing	US	Abnormal	Likely pathogenic
c.841T>C (p.W281R)	PKD, BFIS, ICCA	0	0	0	Deleterious	Probably damaging	Disease causing	US	Abnormal	Likely pathogenic
c.859G>A (p.A287T)	PKD	0	0	0	Deleterious	Possibly damaging	Polymorphism	US	Abnormal	Likely pathogenic
c.872C>T (p.A291V)	PKD	0	0	0	Deleterious	Possibly damaging	Disease causing	US	Abnormal	Likely pathogenic
c.884G>A (p.R295Q)	PKD, PNKD, Migraine	0	0	0	Deleterious	Probably damaging	Disease causing	US	Abnormal	Likely pathogenic
c.913G>A (p.G305R)^d^	PKD, EA, IC	0	8.60E‐06	0	Deleterious	Probably damaging	Disease causing	US	Normal	US
c.913G>T (p.G305W)	PKD	0	0	0	Deleterious	Probably damaging	Disease causing	US	Abnormal	Likely pathogenic
c.916G>A (p.A306T)	BFIS	0	0	0	Deleterious	Probably damaging	Disease causing	US	Abnormal	Likely pathogenic
c.917C>A (p.A306D)	PKD	0	0	0	Deleterious	Probably damaging	Disease causing	US	Abnormal	Likely pathogenic
c.922C>T (p.R308C)	PKD	0	0	0	Deleterious	Probably damaging	Disease causing	US	Abnormal	Likely pathogenic
c.931C>T (p.R311W)^d^	PKD, EA	0	0.0001024	0	Deleterious	Probably damaging	Disease causing	US	Normal	US
c.950G>A (p.S317N)	ICCA	0	0	0	Deleterious	Probably damaging	Disease causing	US	Abnormal	Likely pathogenic
c.967G>A (p.G323R)	PKD	0	0	0	Deleterious	Probably damaging	Disease causing	US	Abnormal	Likely pathogenic
c.968G>A (p.G323E)	BFIS	0	0	0	Deleterious	Probably damaging	Disease causing	US	Abnormal	Likely pathogenic
c.970G>A (p.G324R)	ICCA	0	0	0	Deleterious	Probably damaging	Disease causing	US	Abnormal	Likely pathogenic
c.971G>A (p.G324E)	BFIS	0	0	0	Deleterious	Probably damaging	Disease causing	US	Abnormal	Likely pathogenic
c.981C>G (p.I327M)	PKD, BFIS	0	0	0.000177	Deleterious	Probably damaging	Disease causing	US	Normal	US

Abbreviations: 1000G, 1000Genomes Projects; BECTS, Benign epilepsy with centrotemporal spikes; BFIS, Benign familial infantile seizures; Control, 2800 healthy individuals of Chinese ancestry; DS, Dravet syndrome; EA, Episodic ataxia; ExAc, Exome Aggregation Consortium; FS+, Febrile seizures plus; GEFS+, Generalized epilepsy with febrile seizures plus; IC, Infantile convulsion; ICCA, Infantile convulsions and choreoathetosis; ID, Intellectual Disability; PHD, Paroxysmal hypnogenic dyskinesia; PKD, Paroxysmal kinesigenic dyskinesia; PNKD, Paroxysmal nonkinesiogenic dyskinesia; US, Uncertain significance.

a Missense variants were preliminarily classified without the functional data according to ACMG guidelines.

b The decreased or abnormally localized mutant protein was considered functionally impaired.

c Compound heterozygous missense variant in *PRRT2* had been observed.

d Homozygous missense variant in *PRRT2* had been observed.

Two variants (p.P138A and p.D147H) had a minor allele frequency (MAF) ≥0.05 in a population database^17^ and were predicted to be benign by functional software (Table 1). Another variant (p.P216L) was found to possess 5.2% of 115 controls in an Australian study,^18^ although it was predicted to be deleterious (Table 1). Of note, these three variants were not co‐segregated with the disease in the family pedigrees reported previously.^17^ In addition, eight variants (p.R266W, p.S275F, p.A291V, p.G305R, p.A306T, p.S317N, p.G324E, and p.I327M) were reported to co‐segregate with PKD or BFIS in multiple affected family members.^18^, ^19^, ^20^, ^21^, ^22^, ^23^, ^24^ They were predicted to be deleterious by SIFT, Polyphen‐2, and MutationTaster. All the eight variants but one (p.G305R) were absent in population database and our control. However, they were classified as uncertain significance variants for lack of sufficient evidence. Other 18 variants were also classified as uncertain significance variants combining the population MAF and prediction data.

### 3.2 Missense variants decreased the protein level

**Figure 1:**
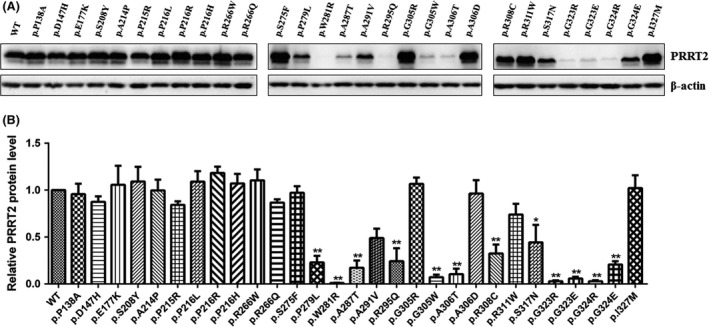
Protein levels of wild‐type and mutant *PRRT2*. A, Western blot analysis of protein extracts obtained from HEK293 cells transfected with pEGFP‐PRRT2 wild‐type or mutant vectors. The anti‐GFP antibody was used to detect the PRRT2 protein. B, Values represent mean ± SE, n = 3. *P < 0.05, **P < 0.01 vs wild‐type

For these variants with uncertain significance, functional experiments were required. To determine the consequence of amino acid change in PRRT2, the expression level of mutant PRRT2 was evaluated in mammalian cells. HEK293T cells were transiently transfected with N‐terminal EGFP tagged wild‐type or mutant *PRRT2*. The protein expression levels were examined by Western blot. The data revealed that 12 variants (p.P279L, p.W281R, p.A287T, p.R295Q, p.G305W, p.A306T, p.R308C, p.S317N, p.G323R, p.G323E, p.G324R, and p.G324E) had a dramatic reduced expression or hardly detectable protein level compared to wild‐type PRRT2 (Figure 1). The remaining 17 variants, including the benign ones, had undifferentiated expression of PRRT2 protein as wild‐type (Figure 1).

### 3.3 Missense variants affected plasma membrane localization

**Figure 2:**
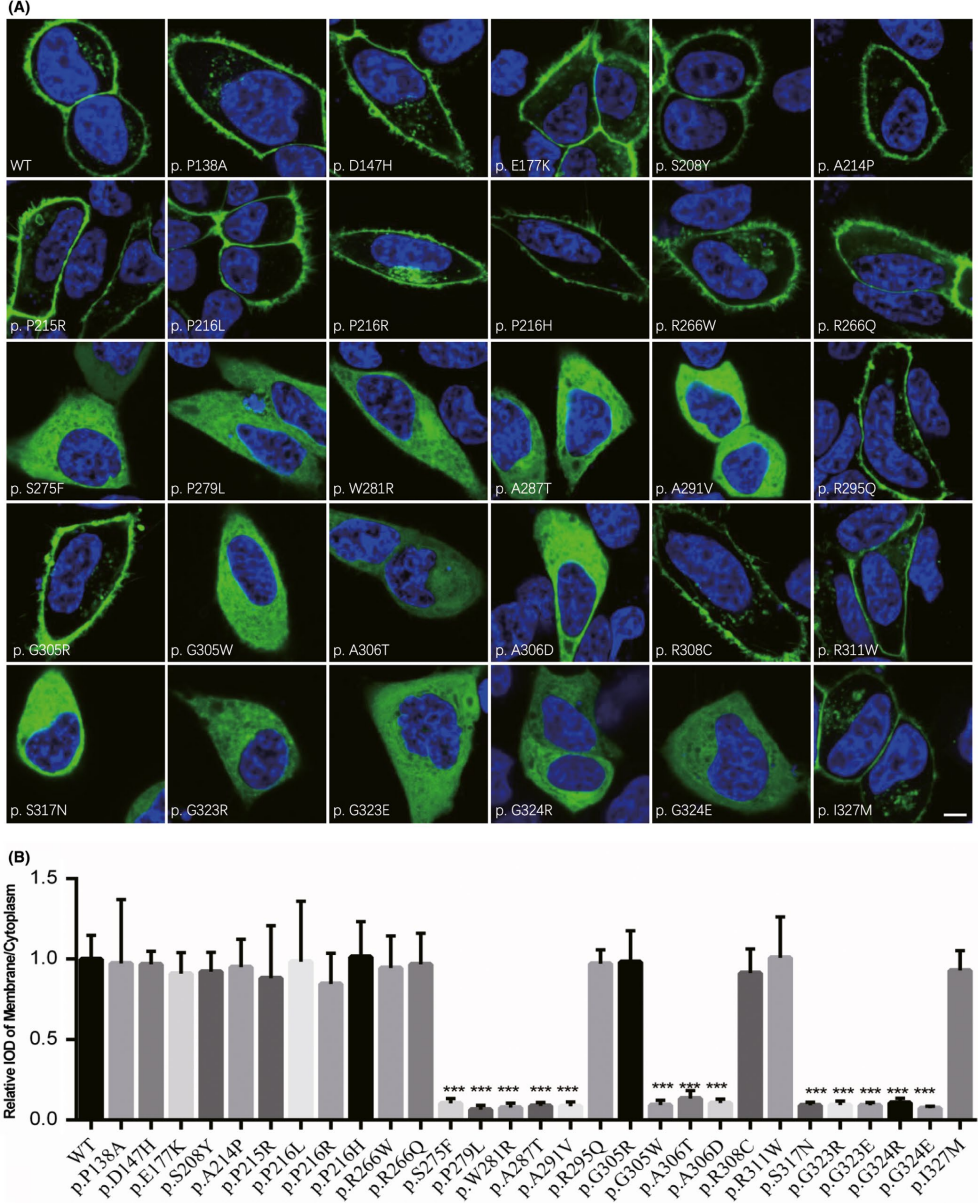
Localization of wild‐type and mutant *PRRT2*. A, HeLa cells transfected with wild‐type and mutant *PRRT2* vectors were examined for green fluorescence 48 h after transfection by a confocal microscope. Cell nuclei were stained with NucBlue Live Reagent (blue). Scale bar, 5 μm. B, The relative IOD ratio of membrane to cytoplasm was calculated by five cells for each variant. Values represent mean ± SE. ***P < 0.001 vs wild‐type

To further characterized the subcellular distribution of mutant PRRT2 harboring missense variants, HeLa cells were transiently transfected with N‐terminal EGFP tagged wild‐type or mutant PRRT2 expression plasmids. Under live cell confocal microscopy, we found that wild‐type PRRT2 was predominantly localized in the plasma membrane, consistent with previously studies.^1^ In contrast, 13 mutant PRRT2 (p.S275F, p.P279L, p.W281R, p.A287T, p.A291V, p.G305W, p.A306T, p.A306D, p.S317N, p.G323R, p.G323E, p.G324R, and p.G324E) lost membrane targeting and were located in the cytoplasm (Figure 2A,B), indicating the alternation of subcellular localization of these missense variants. The remaining 16 variants were still retained in plasma membrane (Figure 2A,B). The red‐fluorescent labeling plasma membrane was shown in the Figure S1.

As expected, the three benign variants (p.P138A, p.D147H, and p.P216L) behaved normally in the *in vitro* functional analysis. Most of uncertain significance variants (15/26) had reduced protein expression or alteration of plasma membrane localization, suggesting functional impairment of these variants. Among them, 10 variants had abnormal both protein expression and intracellular localization, three variants only affected membrane localization, and two variants only affected the protein level.

### 3.4 Classification of the missense variants in *PRRT2*

Decreased protein expression or alternation of subcellular localization was considered functionally impaired. We assigned the pathogenicity of the reported missense variants of *PRRT2* with functional data. As a result (Table 1), 15 variants (p.S275F, p.P279L, p.W281R, p.A287T, p.A291V, p.R295Q, p.G305W, p.A306T, p.A306D, p.R308C, p.S317N, p.G323R, p.G323E, p.G324R, and p.G324E) were classified as “likely pathogenic variants”, 3 (p.P138A, p.D147H, and p.P216L) as “benign variants”, 3 (p.S208Y, p.A214P, and p.P215R) as “likely benign variants” and 8 (p.E177K, p.P216R, p.P216H, p.R266W, p.R266Q, p.G305R, p.R311W, and p.I327M) as “uncertain significance”.

**Figure 3:**
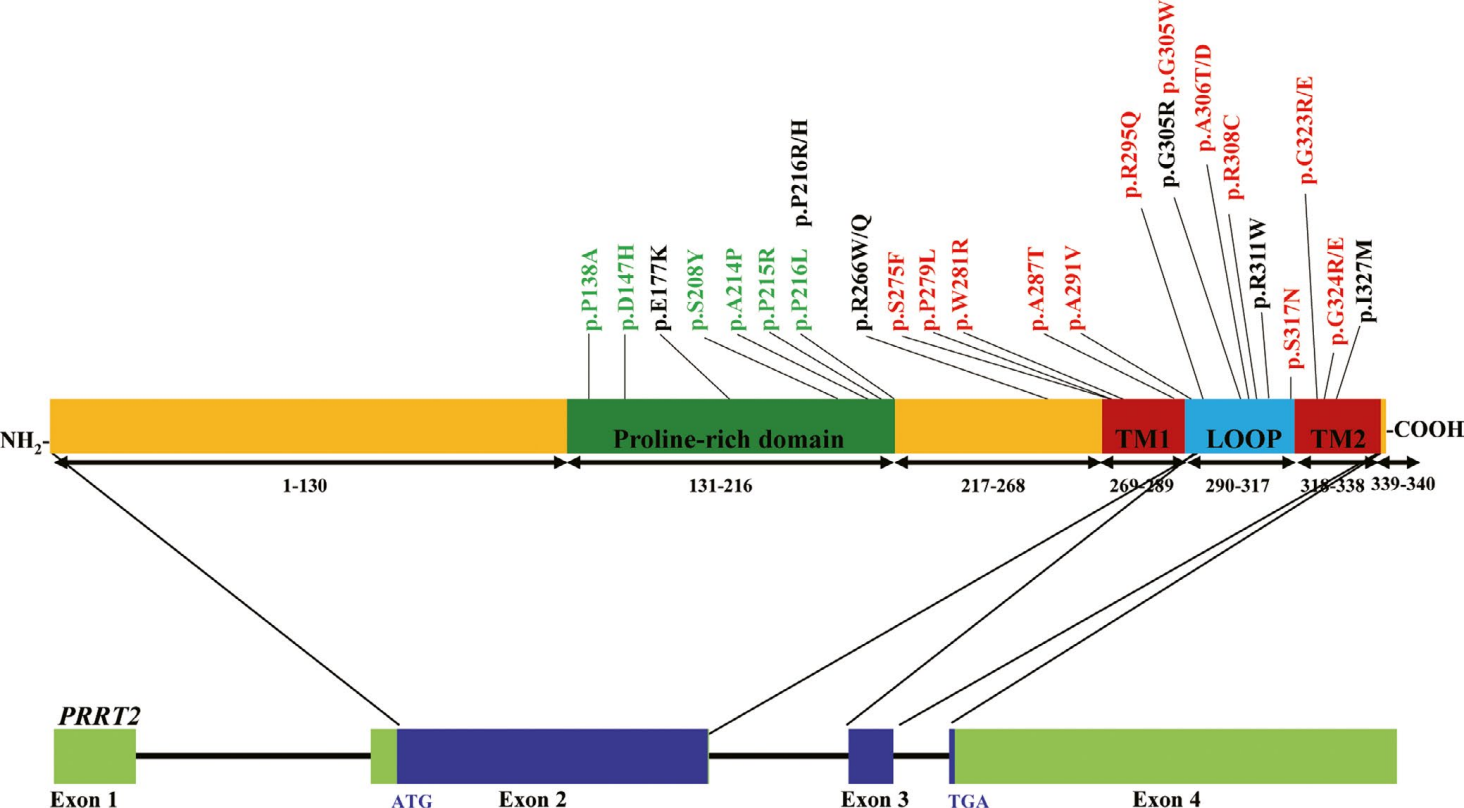
Diagram of the PRRT2 and missense variants. *PRRT2* contains four exons and encodes 340 amino acid residues. The locations of known missense variants in the gene are showed. The likely pathogenic variants are indicated in red. The benign and likely benign variants are in green. The uncertain significance variants are in black

A graphical representation of amino acid change in *PRRT2* was shown in Figure 3. We found that misssense variants were clustered in exon 2 and exon 3 of *PRRT2*. The likely pathogenic variants marked in red were concentrated in the C‐terminal of PRRT2.

## 4 DISCUSSION

*PRRT2* variants are associated with various paroxysmal disorders, implying a common pathway may be involved in these disorders. Thus, it is specifically necessary to elucidate the potential mechanisms of *PRRT2* variants. In addition to frameshift and nonsense mutations, 29 missense variants were also documented in *PRRT2*‐related disorders. Lack of functional experiments in majority of the missense variants makes their pathogenicity uncertain. In this study, we systematically reviewed the *PRRT2*‐related disorders and summarized the reported *PRRT2* missense variants in the literature. We performed functional experiments to investigate the subcellular localization and protein expression of 29 *PRRT2* missense variants. Ten variants were found to affect both plasma membrane localization and protein level, three variants only affect membrane localization, and two variants only affect the protein level. Combining the population MAF, prediction, segregation data, and functional experiments, we classified these missense variants, respectively. To our knowledge, this is the first study to systematically evaluate the significance of *PRRT2* missense variants according to the ACMG guidelines.

Previous studies revealed that *PRRT2* variants modified the phenotype of PKD and caused earlier age at onset, longer duration, and higher frequency of complicated PKD attacks.^7^, ^25^ Although the potential mechanism of *PRRT2* variants in PKD was largely unknown, loss‐of‐function leading to haploinsufficiency was believed to play a crucial role in the pathogenesis. Recently, several studies uncovered the pathophysiological mechanism of PRRT2.^8^, ^11^, ^26^, ^27^ It was demonstrated that PRRT2 was a transmembrane protein localizing primarily to the presynaptic terminals. PRRT2 involved in synaptic vesicle fusion and the release of neurotransmitters through interacting with the synaptic proteins SNAP25 and synaptotagmin 1/2.^8^, ^11^ Moreover, electrophysiological study showed that PRRT2 controlled the excitability of excitatory neurons by interacting with Nav1.2/Nav1.6 channels.^27^

It is widely known that the membrane localization of PRRT2 is crucial for its physiological function. We found 51.7% (15/29) of the *PRRT2* missense variants affected subcellular localization and/or protein level, which was consistent with the hypothesis of loss‐of‐function in *PRRT2*‐related diseases. The reduced protein level of mutant protein p.R308C and the cytoplasma location of mutant protein p.A287T has also been reported previously.^12^, ^13^ The p.G305W has been reported to disrupt the SNARE‐modulatory function.^11^ In our study, p.G305W was also functionally impaired by abnormal subcellular localization and protein level. 20.7% (6/29) of variants were benign or likely benign variants. 27.6% (8/29) of the variants were uncertain significance variants and their pathogenicity needs further investigation. New disease‐causing genetic mutations are waiting to be discovered in these patients with benign *PRRT2* variants or without *PRRT2* variants.^28^

The majority of reported PRRT2 variants are frameshift variants and cause truncation of PRRT2 protein, indicating the crucial role of the C‐terminal. In this study, we found all the likely pathogenic missense variants were localized in the TM domains and loop domain of C‐terminal, while the benign or likely benign missense variants were localized in the N‐terminal. These findings further confirmed the importance of C‐terminal of PRRT2 protein. PRRT2 mutant lacking the large N‐terminal domain was localized correctly to the plasma membrane.^29^ In our study, only the C‐terminal amino acid change could result in mislocalization. Possibly, membrane orientation of PRRT2 is imposed by the C‐terminal, especially the TM domain.

In conclusion, a total of PRRT2 missense variants reported is firstly assessed by the ACMG guidelines. It will be of great value for its instructive and meaningful role in clinical molecular diagnosis. Missense variants could decrease the protein level and/or impair plasma membrane localization. C‐terminal of PRRT2 is crucial for its physiological function. Functional study is vital for the classification and potential mechanisms associated with PRRT2 should be further explored.

## CONFLICT OF INTEREST

The authors declare no conflict of interest.
