# Supplementary material for: Functional study and pathogenicity classification of PRRT2 missense variants in PRRT2‐related disorders
Source: CNS Neurosci Ther. 2019 May 23;26(1):39–46. doi: 10.1111/cns.13147 (PMC6930815; doi:10.1111/cns.13147)
Supplement: Supplementary file 2 [file CNS-26-39-s002.docx]

**Supplementary Table The primers for mutagenesis plasmids**

| **Variant** | **Primers** |
| --- | --- |
| c.412C>G (p.P138A) | F: CCAGCCCCAGAGGCTGCTCCCCAAC |
|  | R: GTTGGGGAGCAGCCTCTGGGGCTGG |
| c.439G>C (p.D147H) | F: GACCCCCGGCCACATTCCCAGCCTA |
|  | R: TAGGCTGGGAATGTGGCCGGGGGTC |
| c.529G>A (p.E177K) | F: TGTCTGAGAGTGTAGGGAAAAAGCAAGAGAATGGG |
|  | R: CCCATTCTCTTGCTTTTTCCCTACACTCTCAGACA |
| c.623C>A (p.S208Y) | F: TGGCTGGGGGGTATTTTTTTGAGGGTGGTGAGT |
|  | R: ACTCACCACCCTCAAAAAAATACCCCCCAGCCA |
| c.640G>C (p.A214P) | F: TCGGGGGGGGGGCCCATTGGCTG |
|  | R: CAGCCAATGGGCCCCCCCCCCGA |
| c.644C>G (p.P215R) | F: GCACTCGGGGGCGGGCCCCATTG |
|  | R: CAATGGGGCCCGCCCCCGAGTGC |
| c.647C>T (p.P216L) | F: TGGGGCCCCCCTCCGAGTGCTGC |
|  | R: GCAGCACTCGGAGGGGGGCCCCA |
| c.647C>G (p.P216R) | F: GGGGCCCCCCGCCGAGTGCTG |
|  | R: CAGCACTCGGCGGGGGGCCCC |
| c.647C>A (p.P216H) | F: CCAATGGGGCCCCCCACCGAGTG |
|  | R: CACTCGGTGGGGGGCCCCATTGG |
| c.796C>T (p.R266W) | F: GTGAAGGCACCCAGAAACCTTGGGACTACATCATC |
|  | R: GATGATGTAGTCCCAAGGTTTCTGGGTGCCTTCAC |
| c.797G>A (p.R266Q) | F: GGCAAGGATGATGTAGTCCTGAGGTTTCTGGGTGCCTTC |
|  | R: GAAGGCACCCAGAAACCTCAGGACTACATCATCCTTGCC |
| c.824C>T (p.S275F) | F: CCACATGGGGCAGAAGCAGAACAGGATGGCAAGGATGATG |
|  | R: CATCATCCTTGCCATCCTGTTCTGCTTCTGCCCCATGTGG |
| c.836C>T (p.P279L) | F: GTCCTGCTTCTGCCTCATGTGGCCTGTCA |
|  | R: TGACAGGCCACATGAGGCAGAAGCAGGAC |
| c.841T>C (p.W281R) | F: GATGTTGACAGGCCGCATGGGGCAGAAGC |
|  | R: GCTTCTGCCCCATGCGGCCTGTCAACATC |
| c.859G>A (p.A287T) | F: ACAGCATAAGCGAAGGTCACGATGTTGACAGGC |
|  | R: GCCTGTCAACATCGTGACCTTCGCTTATGCTGT |
| c.872C>T (p.A291V) | F: GTTCCGGGACATGACAACATAAGCGAAGGCCAC |
|  | R: GTGGCCTTCGCTTATGTTGTCATGTCCCGGAAC |
| c.884G>A (p.R295Q) | F: TGCTGTCATGTCCCAGAACAGCCTGCAGC |
|  | R: GCTGCAGGCTGTTCTGGGACATGACAGCA |
| c.913G>A (p.G305R) | F: GGGACGTGGACAGGGCCCAGCGT |
|  | R: ACGCTGGGCCCTGTCCACGTCCC |
| c.913G>T (p.G305W) | F: GGGACGTGGACTGGGCCCAGCGT |
|  | R: ACGCTGGGCCCAGTCCACGTCCC |
| c.916G>A (p.A306T) | F: GACGTGGACGGGACCCAGCGTCTGG |
|  | R: CCAGACGCTGGGTCCCGTCCACGTC |
| c.917C>A (p.A306D) | F: CCAGACGCTGGTCCCCGTCCACG |
|  | R: CGTGGACGGGGACCAGCGTCTGG(weidui) |
| c.922C>T (p.R308C) | F: CCGGCCCAGACACTGGGCCCCGT |
|  | R: ACGGGGCCCAGTGTCTGGGCCGG |
| c.931C>T (p.R311W) | F: CAGCGTCTGGGCTGGGTAGCCAAGC |
|  | R: GCTTGGCTACCCAGCCCAGACGCTG |
| c.950G>A (p.S317N) | F: GTAGCCAAGCTCTTAAACATCGTGGCGCTGG |
|  | R: CCAGCGCCACGATGTTTAAGAGCTTGGCTAC |
| c.967G>A (p.G323R) | F: GTGGCGCTGGTGAGGGGAGTCCTCA |
|  | R: TGAGGACTCCCCTCACCAGCGCCAC |
| c.968G>A (p.G323E) | F: GTGGCGCTGGTGGAGGGAGTCCTCATC |
|  | R: GATGAGGACTCCCTCCACCAGCGCCAC |
| c.970G>A (p.G324R) | F: ATGATGAGGACTCTCCCCACCAGCGCC |
|  | R: GGCGCTGGTGGGGAGAGTCCTCATCAT |
| c.971G>A (p.G324E) | F: GCGCTGGTGGGGGAAGTCCTCATCATC |
|  | R: GATGATGAGGACTTCCCCCACCAGCGC |
| c.981C>G (p.I327M) | F: GGGGGGAGTCCTCATGATCATCGCCTCC |
|  | R: GGAGGCGATGATCATGAGGACTCCCCCC |
