# Supplementary material for: Functional study and pathogenicity classification of PRRT2 missense variants in PRRT2‐related disorders
Source: CNS Neurosci Ther. 2019 May 23;26(1):39–46. doi: 10.1111/cns.13147 (PMC6930815; doi:10.1111/cns.13147)
Supplement: Supplementary file 3 [file CNS-26-39-s003.docx]

**Supplementary Figure Localization of wild-type and mutant *PRRT2.*** HeLa cells transfected with wild-type and mutant *PRRT2* vectors were examined for green fluorescence 48 hours after transfection by a confocal microscope. Plasma membrane was stained by red fluorescent WGA. Scale bar, 5 μm.
